# Supplementary material for: Linking ecology and systematics of acidobacteria: Distinct habitat preferences of the Acidobacteriia and Blastocatellia in tundra soils
Source: PLoS One. 2020 Mar 17;15(3):e0230157. doi: 10.1371/journal.pone.0230157 (PMC7077872; doi:10.1371/journal.pone.0230157)
Supplement: S1 Table — (PDF) [file pone.0230157.s001.pdf]

S1 Table. Relative abundance of different acidobacterial groups (means ± standard deviation).

**Site1**	**US**	**SF**	**MS**
Acidobacteriia;Acidobacteriales Acidobacteriaceae	0,00±0,01	0,11±0,04	0,21±0,02
Acidobacteriia;Acidobacteriales uncultured	0,35±0,07	0,29±0,08	0,33±0,06
Acidobacteriia;Bryobacterales	0,12±0,04	0,36±0,09	0,17±0,06
Acidobacteriia;SD2	0,07±0,08	0,18±0,03	0,22±0,07
Blastocatellia (SD4)	0,31±0,02	0,04±0,02	0,07±0,04
Holophagae;SD7	0,12±0,01	0,02±0,01	0,00±0,01
Other	0,02±0,01	0,00±0,00	0,00±0,00
**Site2**			
Acidobacteriia;Acidobacteriales Acidobacteriaceae	0,13±0,05	0,27±0,08	0,32±0,08
Acidobacteriia;Acidobacteriales uncultured	0,29±0,07	0,18±0,07	0,25±0,05
Acidobacteriia;Bryobacterales	0,33±0,06	0,50±0,04	0,22±0,04
Acidobacteriia;SD2	0,04±0,02	0,02±0,01	0,20±0,11
Blastocatellia (SD4)	0,14±0,05	0,03±0,02	0,00±0,00
Holophagae;SD7	0,07±0,03	0,00±0,00	0,00±0,00
Other	0,02±0,03	0,00±0,00	0,01±0,01
