# Supplementary material for: Linking ecology and systematics of acidobacteria: Distinct habitat preferences of the Acidobacteriia and Blastocatellia in tundra soils
Source: PLoS One. 2020 Mar 17;15(3):e0230157. doi: 10.1371/journal.pone.0230157 (PMC7077872; doi:10.1371/journal.pone.0230157)
Supplement: S2 Table — (PDF) [file pone.0230157.s002.pdf]

S2 Table. Statistical values calculated by multiple t-test comparisons for acidobacterial groups from Site 1 and 2. Statistically significant values (P-value confidence level<0.05) are indicated by bold.

Taxa	Site1			Site2		
	US/SF	SF/MS	US/MS	US/SF	SF/MS	US/MS
	P-value	P-value	P-value	P-value	P-value	P-value
Acidobacteriia;Acidobacteriales Acidobacteriaceae	**0,00659**	0,121839	**< 0,0001**	**0,000139**	0,284717	**< 0,0001**
Acidobacteriia;Acidobacteriales uncultured	0,153357	0,121839	> 0,9999	**0,005011**	0,110811	0,308411
Acidobacteriia;Bryobacterales	**< 0,0001**	**< 0,0001**	0,409868	**0,000139**	**< 0,0001**	0,012865
Acidobacteriia;SD2	**0,00659**	0,431744	**0,000257**	0,561333	**< 0,0001**	**0,00013**
Blastocatellia (SD4)	**< 0,0001**	0,431744	**< 0,0001**	**0,000903**	0,591296	**0,00068**
Holophagae;SD7	0,036116	> 0,9999	0,01813	0,085067	> 0,9999	0,128738
Other	> 0,9999	> 0,9999	> 0,9999	0,561333	> 0,9999	0,609305
