# Supplementary material for: Linking ecology and systematics of acidobacteria: Distinct habitat preferences of the Acidobacteriia and Blastocatellia in tundra soils
Source: PLoS One. 2020 Mar 17;15(3):e0230157. doi: 10.1371/journal.pone.0230157 (PMC7077872; doi:10.1371/journal.pone.0230157)

S1 Figure. Venn diagrams showing the number of shared and unique OTUs in Sites 1 and 2, and in three types of successional stages examined in this study.

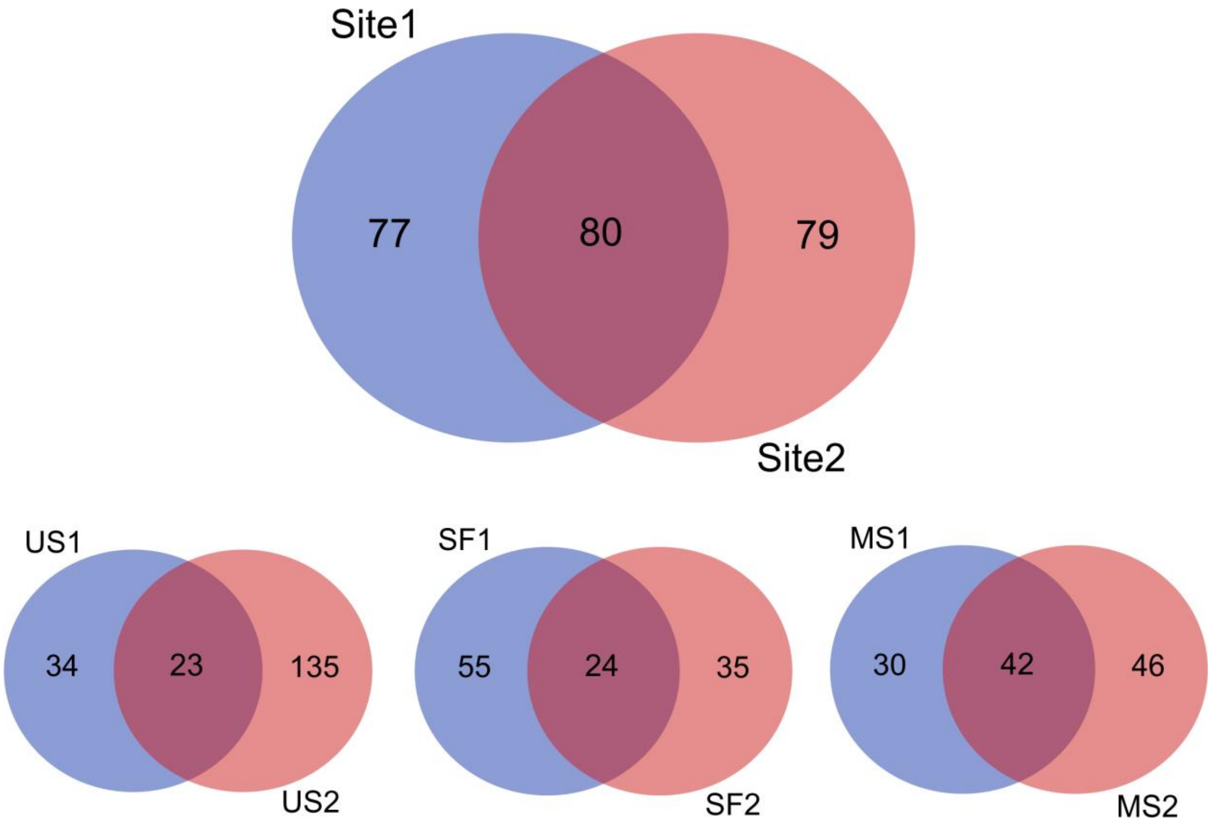

Supplement: S1 Fig — (PDF) [file pone.0230157.s003.pdf]
