# Supplementary material for: Linking ecology and systematics of acidobacteria: Distinct habitat preferences of the Acidobacteriia and Blastocatellia in tundra soils
Source: PLoS One. 2020 Mar 17;15(3):e0230157. doi: 10.1371/journal.pone.0230157 (PMC7077872; doi:10.1371/journal.pone.0230157)

S2 Figure. Heat map showing the relative abundances of well-represented acidobacterial OTUs detected in tundra soils along a gradient of increasing vegetation. The digits in the different boxes indicate the percentage abundance of the particular OTU related to the total number of bacterial ribo-tags affiliated with the *Acidobacteria* in a particular sample.

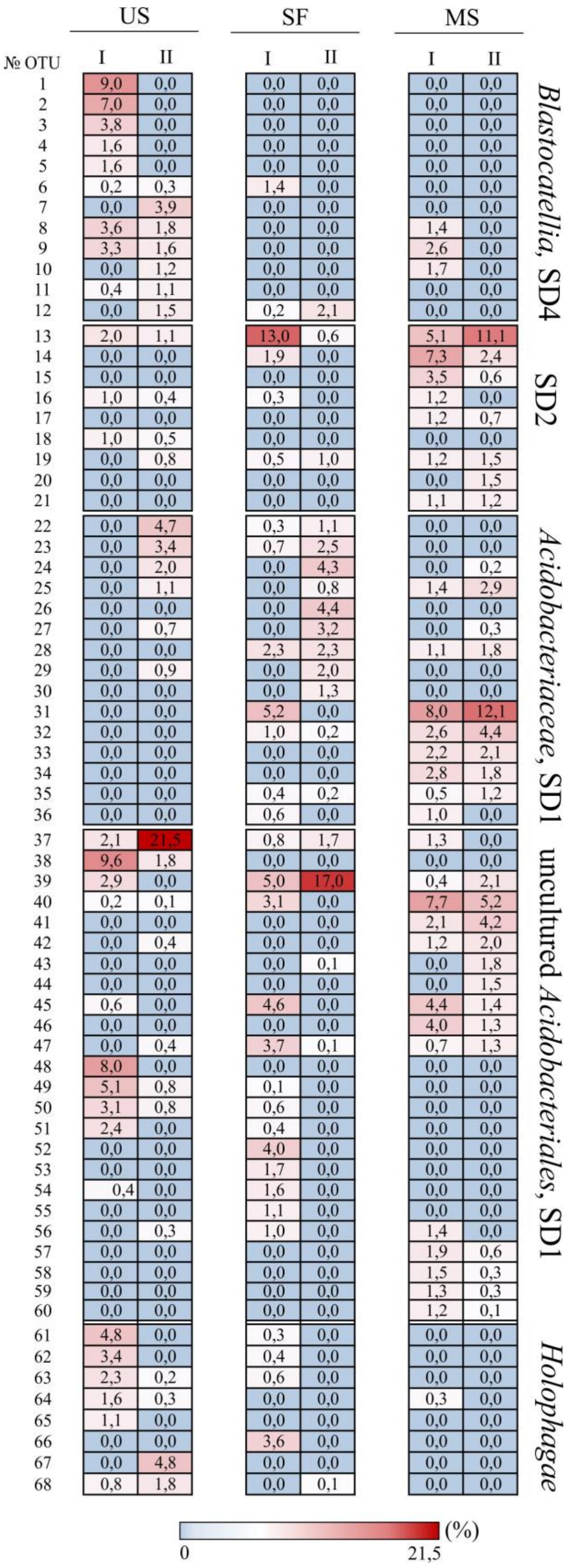

Supplement: S2 Fig — (PDF) [file pone.0230157.s004.pdf]
