# Supplementary material for: Linking ecology and systematics of acidobacteria: Distinct habitat preferences of the Acidobacteriia and Blastocatellia in tundra soils
Source: PLoS One. 2020 Mar 17;15(3):e0230157. doi: 10.1371/journal.pone.0230157 (PMC7077872; doi:10.1371/journal.pone.0230157)
Supplement: Attachment — Submitted filename: Review_PONE-D-19-23462.docx [file pone.0230157.s005.docx]

**Linking ecology and systematics of acidobacteria: Distinct habitat preference of the Acidobacteeria and Blastocatellia in Tundra soils (PONE-D-19-23462**

**Reviewed by Dr. Ali Quoreshi**

**Comments to Author**

In general, the manuscript is well-written and has scientific merit. There are few minor corrections needed to be revised. The research tried to reveal ecological preferences of traits of acidobacteria from different classes form a gradient of sandy soils of tundra. Indeed an interesting study.

In materials and methods section (lines 102-109), it is not very clear to me about the sample size, replicates. Please provide little more details about the sampling protocol, actual numbers of true replicate from each site, is there any composite samples taken? If so then please provide details.

If I don’t misunderstand, the table 3 and Figure 3 are showing very similar information. Author may consider to adding Figure 3 in the supplementary section.

The discussion is well written. I have few concerns.

Lines 277-280, author mentioned about a shift of bacterial community and nicely shown in Figure 7. Please provide some discussions about why these shifts observed.

Authors have discussed current results and compared many other studies and appropriate references were cited. I suggest authors discussing and comparing little more in microbial compositions between the current study results and results from other ecosystems/desert/boreal/rain forest.

Table 4, please add heading under the bacterial groups listed.

Author should try to provide a better quality picture of Figure 1.

Finally, please revise the conclusions. I would like to see more relevant conclusions based on the key results and what described in the abstract.

Good luck.
